# Optimal Configuration for Relaxation Times Estimation in Complex Spin Echo Imaging

**DOI:** 10.3390/s140202182

**Published:** 2014-01-28

**Authors:** Fabio Baselice, Giampaolo Ferraioli, Alessandro Grassia, Vito Pascazio

**Affiliations:** 1 Dipartimento di Ingegneria, Università degli Studi di Napoli Parthenope, Napoli 80143, Italy; E-Mails: alessandro.grassia@uniparthenope.it (A.G.); vito.pascazio@uniparthenope.it (V.P.); 2 Dipartimento di Scienze e Tecnologie, Università degli Studi di Napoli Parthenope, Napoli 80143, Italy; E-Mail: giampaolo.ferraioli@uniparthenope.it

**Keywords:** Magnetic Resonance Imaging, complex decomposition, statistical signal processing, Cramer, Rao lower bounds, relaxation time estimation

## Abstract

Many pathologies can be identified by evaluating differences raised in the physical parameters of involved tissues. In a Magnetic Resonance Imaging (MRI) framework, spin-lattice *T*_1_ and spin-spin *T*_2_ relaxation time parameters play a major role in such an identification. In this manuscript, a theoretical study related to the evaluation of the achievable performances in the estimation of relaxation times in MRI is proposed. After a discussion about the considered acquisition model, an analysis on the ideal imaging acquisition parameters in the case of spin echo sequences, *i.e.*, echo and repetition times, is conducted. In particular, the aim of the manuscript consists in providing an empirical rule for optimal imaging parameter identification with respect to the tissues under investigation. Theoretical results are validated on different datasets in order to show the effectiveness of the presented study and of the proposed methodology.

## 1. Introduction

Relaxation times define the rate of spin magnetic equilibrium recovery in nuclear magnetic resonance (NMR) [1,2]. For each tissue, several relaxation times can be defined. Besides, the main interest is in the evaluation of two of them: the spin-lattice and the spin-spin relaxation times, commonly referred to as *T*_1_ and *T*_2_, respectively. Such time constants, together with the hydrogen nuclei abundance, *ρ*, define the behavior of the signal generated by each resolution element.

It is largely known that the knowledge of relaxation times can provide interesting information about imaged tissues. Concerning the medical diagnostic field, many pathologies have been found to involve a significant variation of the relaxation time constants more than a variation of *ρ*, such as Alzheimer's disease [3], Parkinson's disease [4] and cancer [5,6]. The evaluation of the tissue relaxation times can be considered an excellent tool for improving clinical diagnosis.

Classic approaches for retrieving relaxation parameter maps of imaged tissue slices propose the estimation of *T*_1_ and *T*_2_ separately. In particular, the “gold standard” for spin-lattice relaxation time *T*_1_ estimation exploits inversion recovery (IR) sequences [7,8]. However, this approach is too slow for *in vivo* clinical applications. Different evolutions have been proposed in the literature. In particular, the exploitation of spoiled gradient-recalled echo (SPGR) sequences has shown interesting results [9,10]. With respect to spin-spin relaxation time *T*_2_ estimation, a widely used imaging sequence is the spin echo (SE) [11,12].

The magnitude of the acquired signal is typically used for relaxation parameter estimation [12–15]. Within this framework, the exponential curve fitting via the least squares (LS) algorithm is the commonly adopted estimator [11,13]. Although being very easy to be implemented and not computationally heavy, it has the disadvantage of producing biased estimations [11,16]. The alternative consists in using a maximum likelihood estimator (MLE) [12]. The MLE is asymptotically unbiased and optimal, but the function to be maximized, which is related to the statistical distribution of the MRI amplitude data, is computationally heavy, as it contains the Bessel function [17].

Recently, new approaches based on the complex decomposition of acquired data have been proposed [10,18]. The exploitation of the complex model leads to a main advantage concerning the estimation: due to the circular Gaussian distribution of the complex noise, the LS-based estimator coincides with the MLE and is asymptotically unbiased and optimal.

While much effort has been directed to improving the estimation procedures, only a little effort has been directed to the choice of the optimal imaging parameter selection (*i.e.*, the optimal choice of the MRI scanner imaging parameters). In particular, in [19], the ideal repetition times have been investigated in the case of saturation recovery spin-lattice measurements at 4.7 T, while in [20], the optimization of *T*_2_ measurements in the case of bi-exponential systems is considered. Following the approach proposed by [15], within this paper we investigate the possibility of finding the optimal imaging parameter configuration for relaxation time estimation. As an alternative to [15], we investigate the optimal configuration not only for the *T*_2_ time estimation, but for the joint *T*_2_ and *T*_1_ estimation.

Since the SE sequence-based model allows the simultaneous estimation of both spin-spin and spin-lattice relaxation times, we focus our attention on this imaging sequence. In any case, the theoretical study reported in the following could be easily adapted to different imaging sequences. Considering an SE sequence [2], the two imaging parameters involved in the acquisition procedure are the repetition time, *T_R_*, and the echo time, *T_E_*. We briefly recall that these two parameters allow the scanner to differently interact with tissues characterized by different *T*_1_ and *T*_2_ values. By exploiting different *T_R_* and *T_E_* combinations, it is possible to emphasize the effect of one tissue intrinsic parameter with respect to others, obtaining the well-known *ρ*-weighted, *T*_1_-weighted or *T*_2_-weighted images.

Given the previously mentioned motivations, we present a deeper analysis of the complex SE model considered in [18] extended to three parameters (*i.e.*, *ρ*, *T*_1_ and *T*_2_). The analysis is conducted exploiting the Cramer–Rao lower bounds (CRLBs) [16]. Since CRLBs provide the best achievable performances in the unbiased estimation of one or more parameters, by minimizing them with respect to the MR scanner imaging parameters, it is possible to find the optimal acquisition configuration for the relaxation time estimation. Practically speaking, we look for the acquisition parameters that allow achieving lower relaxation time estimation errors. The result of the study is the introduction of a general empirical rule for determining the optimal (with respect to CRLBs) MRI scanner parameter configuration. In particular, the identification of these parameters in the case of several tissues has been conducted. The effectiveness of the theoretical results and of the empirical rule is validated and verified on different datasets.

The manuscript is organized as follows: in Section 2, the acquisition model for an MRI spin echo sequence is presented, and in Section 3, the achievable performances of the estimation are analyzed via the CRLBs. In Section 4, the CRLB-based empirical rule for the optimal acquisition parameter configuration is presented. Finally, validation on different datasets is presented in Section 5, and conclusions are drawn.

## 2. The Model

Let us consider an MRI acquisition system using a spin echo imaging sequence. The amplitude of the recorded complex signal after the image formation process, *i.e.*, after the computation of the 2D Fourier transform, is related to the tissue parameters, *ρ*, *T*_1_ and *T*_2_, via [2,21]:

(1) $$f(\theta )=\rho \exp\;\left(-\frac{T_{E}}{T_{2}}\right)\;\left(1-\exp\left(-\frac{T_{R}}{T_{1}}\right)\right)$$

where *T_E_* and *T_R_* are the echo and repetition time, respectively, which are two imaging parameters that can be set in the MRI scanner, and ***θ*** = [*ρ T*_1_ *T*_2_]*^T^* is the vector containing the tissue parameters in which we are interested. Note that Equation (1) is valid in the case of a homogeneous imaged object. In the case of clinical data, the presence of different hydrogen environments within each voxel has to be taken into account. The acquisition model reported in Equation (1), which is a solution to Bloch equations, assuming that *T_E_* is short with respect to *T_R_*, is related to the noise-free case and does not take into account the dependency on the static magnetic field, *B*. Considering noise, in the complex domain, the model becomes:

(2) $$y=y_{R}+iy_{I}=f(\theta )\exp(i\phi )+(n_{R}+in_{I})$$

where *n_R_* and *n_I_* are the real and imaginary parts of the noise samples, which are distributed as independent circularly Gaussian variables [22], and *ϕ* represents the angle of the complex data [23,24]. Thus, the statistical distributions of the real and imaginary parts of the acquired signal are:

$$f_{Y_{R}}(y_{R})=\frac{1}{\sqrt{2\pi \sigma^{2}}}\exp\;\left(-\frac{{\left(y_{R}-f(\theta )\cos(\phi )\right)}^{2}}{2\sigma^{2}}\right)$$

(3) $$f_{Y_{I}}(y_{I})=\frac{1}{\sqrt{2\pi \sigma^{2}}}\exp\;\left(-\frac{{\left(y_{I}-f(\theta )\sin(\phi )\right)}^{2}}{2\sigma^{2}}\right)$$

where *σ*^2^ is the variance of real and imaginary noise components. Due to the independence of the real and imaginary parts of noise, the joint statistical distribution of *y_R_* and *y_I_* is the product of the two probability density functions of Equation (3).

Once *N* acquisitions with different *T_R_* and *T_E_* combinations have been recorded and collected in the data vector ***y*** = [***y****_R_*, ***y****_I_*], with ***y****_R_* = [*y_R_*(1), ⋯ *y_R_*(*N*)] and ***y****_I_* = [*y_I_*(1), ⋯ *y_I_*(*N*)], we can derive the likelihood function from the factorization of the Probability Density Functions (PDFs):

(4) $$p(y;\theta )=\prod_{k=1}^{N}\;{\left(\frac{1}{\sqrt{2\pi \sigma^{2}}}\right)}^{2}\exp\;\left\{-\frac{{\left[y_{R}(k)-f(\theta )\cos(\phi )\right]}^{2}}{2\sigma^{2}}-\frac{{\left[y_{I}(k)-f(\theta )\sin(\phi )\right]}^{2}}{2\sigma^{2}}\right\}$$

Starting from the likelihood function of Equation (4), the CRLBs for *θ* are derived and analyzed in the following sections.

## 3. Cramer-Rao Lower Bounds Evaluations

In order to evaluate the performances of the optimal estimator for the model presented in Section 2, the Cramer–Rao lower bounds have to be computed. According to Statistical Estimation Theory [16], given an observation model, the accuracy of any estimator can be evaluated according to its mean and its variance. In particular, in order to be optimal, an estimator needs to have its mean equal to the value to be estimated (*i.e.*, unbiased estimator) and to have the smallest possible variance. CRLBs represent the lower bound of the variance of any unbiased estimator, resulting an interesting and powerful tool for evaluating the achievable performances of a considered model. By computing the CRLBs for different configuration of the parameters involved in the acquisition model, it is possible to find the best parameter configuration, the one that provides the minimum values of CRLBs (*i.e.*, the minimum achievable variances). Considering the vector parameter ***θ***, the minimum variance that any unbiased estimator of parameter *θ_i_* can reach is provided by the *i*-th diagonal element of the inverse of matrix **I** [16]:

(5) $$\text{var}({\hat{\theta }}_{i})\geq {\left[{\text{I}}^{-1}(\theta )\right]}_{ii}$$

with **I** being the Fisher matrix, which is equal to:

(6) $$\text{I}(\theta )=\left[\begin{array}{lll} -E\;\left[\frac{\partial^{2}\ln\;p(\text{y};\theta )}{\partial \rho^{2}}\right] & -E\;\left[\frac{\partial^{2}\ln\;p(\text{y};\theta )}{\partial \rho \partial T_{1}}\right] & -E\;\left[\frac{\partial^{2}\text{In}\;p(y;\theta )}{\partial \rho \partial T_{2}}\right]\\ -E\;\left[\frac{\partial^{2}\ln\;p(y;\theta )}{\partial \rho \partial T_{1}}\right] & -E\;\left[\frac{\partial^{2}\ln\;p(y;\theta )}{\partial T_{1}^{2}}\right] & -E\;\left[\frac{\partial^{2}\ln\;p(y;\theta )}{\partial T_{1}\partial T_{2}}\right]\\ -E\;\left[\frac{\partial^{2}\ln\;p(y;\theta )}{\partial \rho \partial T_{2}}\right] & -E\;\left[\frac{\partial^{2}\ln\;p(y;\theta )}{\partial T_{1}\partial T_{2}}\right] & -E\;\left[\frac{\partial^{2}\ln\;p(y;\theta )}{\partial T_{2}^{2}}\right] \end{array}\right]$$

where *E* [·] is the expected value operator.

A closed form for the second order derivatives of Equation (6) has been derived and reported in the Appendix. The closed form greatly improves the computational accuracy of the CRLB evaluation and decreases the computational burden of the simulations reported in the following.

In order to experimentally compute the matrix of Equation (6), Monte Carlo simulations with 10^5^ iterations have been considered for statistical average computation.

For the following evaluations, we considered a tissue, named *A*, with parameters ***θ*** = [*ρ T*_1_ *T*_2_]*^T^* = [2.5 1600 90]*^T^*. Note that within the paper, all relaxation times are expressed in milliseconds, while the proton density is in percentage. The following simulations are reported and analyzed in order to investigate CRLB dependency and behavior with respect to the signal-to-noise ratio (SNR), the number of acquisitions and the scanner acquisition parameters.

### 3.1. *CRLB* vs. *SNR*

**Figure 1.**
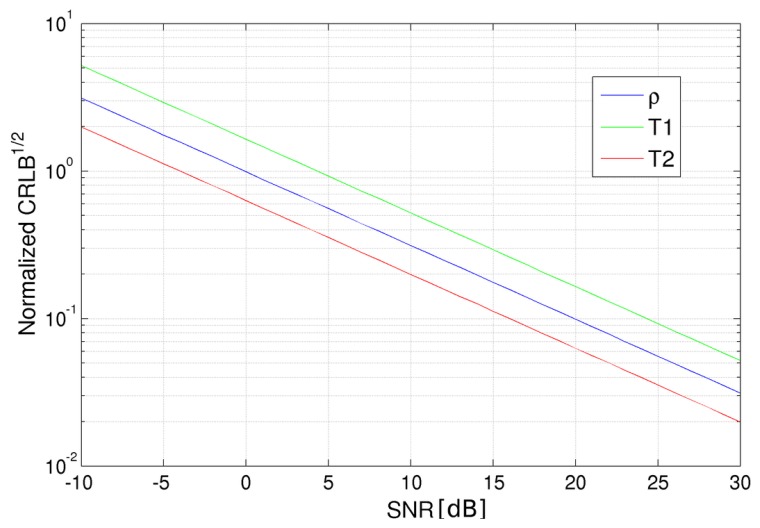
Square root of the Cramer–Rao lower bound (CRLB) for proton density (blue), spin-lattice (*T*_1_) relaxation time (green) and spin-spin (*T*_2_) relaxation time (red) for different signal-to-noise ratio values expressed in decibels (logarithmic scale). CRLB values have been normalized for the parameter values in order to be plotted in the same graph.

Let us start by computing CRLBs varying the noise standard deviation (*i.e.*, the SNR). Sixteen images have been considered, which refer to the all combinations of four *T_R_* and four *T_E_* values equally spaced in the intervals [500, 3500] ms and [80, 350] ms, respectively. Note that the lower *T_E_* value has been set according to the minimum echo time for the SE sequence accepted by the Philips Achieva 3.0 T, the MR scanner we worked on, while the maximum value of *T_R_* has been set in order to limit the global acquisition time. The CRLBs in the case of different SNRs are shown in Figure 1. As expected, the square root of the CRLBs related to all considered parameters decreases with respect to SNR growth, *i.e.*, high SNRs positively affect the estimator performances. In the considered range of SNRs, no saturation appears. Very similar behaviors are obtained varying *T_R_* and *T_E_* combinations. Globally, it can be stated that SNR linearly affects CRLBs, so in the following the results of each simulation can be easily extended to any SNR configuration.

### 3.2. *CRLB* vs. *the Number of Acquisitions*

**Figure 2.**
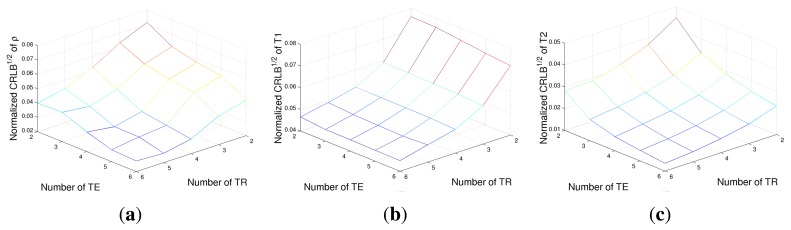
The square root of the CRLB of proton density *ρ* (**a**), *T*_1_ relaxation time (**b**) and *T*_2_ relaxation time (**c**) for different numbers of acquisitions.

A second case study has been conducted in order to evaluate the advantage of increasing the number of acquisitions. Two vectors of *T_R_* and *T_E_*, of a length of *N_R_* and *N_E_*, respectively, have been generated in the [500, 3500] ms (for *T_R_*) and [80, 350] ms (for *T_E_*) intervals. The square root of CRLBs, *i.e.*, the minimum achievable standard deviations, are reported in Figure 2 for *ρ*, *T*_1_ and *T*_2_, respectively, for different *N_R_* and *N_E_* combinations. The noise variance has been fixed in order to obtain an SNR of 16 dB for the image with the lowest signal intensity (*i.e.*, *T_R_* = 500 ms and *T_E_* = 80 ms). It can be noted that the number of *T_R_* values mainly affects the achievable performances with respect to *T*_1_ estimation, while CRLBs of *ρ* and *T*_2_ are dependent on the number of both *T_E_* and *T_R_* values, with a higher dependency on echo times. The results confirm the strict connections between *T_R_* and *T*_1_ and also between *T_E_* and *T*_2_, as expected from the exponential terms of the SE signal model (Equation (1)). However, it is interesting to stress how the CRLB of *ρ* is very tightly related to *T_E_* values rather than to *T_R_* ones.

### 3.3. *CRLB* vs. *T_R_ and T_E_ Values*

**Figure 3.**
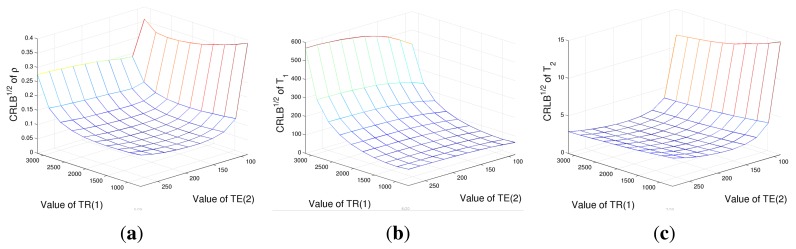
The square root of the CRLB of proton density *ρ* (**a**), *T*_1_ relaxation time (**b**) and *T*_2_ relaxation time (**c**) for different combinations of *T_R_* and *T_E_* values in the case of *ρ* = 2.5, *T*_1_ = 1,600 ms and *T*_2_ = 90 ms.

**Figure 4.**
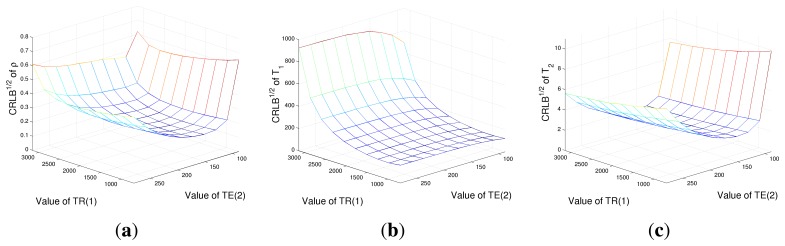
The square root of the CRLB of proton density *ρ* (**a**), *T*_1_ relaxation time (**b**) and *T*_2_ relaxation time (**c**) for different combinations of *T_R_* and *T_E_* values in the case of *ρ* = 2.8, *T*_1_ = 1,800 ms and *T*_2_ = 60 ms.

As a further case study, an evaluation of CRLBs with respect to *T_R_* and *T_E_* values with a fixed number of acquisition has been performed. Four acquisitions have been considered, corresponding to all the combinations of **T***_R_* = [*T_R_*(1), *T_R_*(2)] and **T***_E_* = [*T_E_*(1), *T_E_*(2)]. CRLBs have been computed while varying *T_R_*(1) and *T_E_*(2) and considering *T_R_*(2)= 3, 500 ms and *T_E_*(1) = 80 ms, again in the case of tissue *A* parameters. Results are reported in Figures 3. Figure 4a shows that the *ρ* estimation would prefer low *T_R_* (1) and high *T_E_*(2) values. The behaviors of CRLBs for *T*_1_ and *T*_2_ differ remarkably from Figure 4a, as it can be noticed that the estimation of *T*_1_ is almost unresponsive with respect to *T_E_*(2) values, as far as *T*_2_ estimation with respect to *T_R_*(1). In particular, for the estimation of *T*_1_, the ideal *T_R_*(1) is as low as possible, while the ideal *T_E_*(2) for the estimation of *T*_2_ is between 150 and 250 ms. For this experiment, a second dataset has also been considered: the same simulation has been conducted in the case of a second tissue, named *B*, with parameters ***θ*** = [*ρ T*_1_ *T*_2_] *^T^* = [2.8 1800 60]*^T^*, in order to know if the results of Figure 3 are always valid or if they are highly dependent on the considered tissue. The results are reported in Figure 4. It can be noticed that the lower regions remain in the same position, although being increased in value, but for CRLBs of *ρ* and *T*_2_, the ideal *T_E_*(2) range reduces to [130, 180] ms. This is mainly due to the lower *T*_2_ value of tissue *B* with respect to tissue *A*. Thus, it can be concluded that the general trend is confirmed, although the position of the global minimum is strictly related to the considered tissue. These two simulations show that the choice of optimal parameters is strictly dependent on the relaxation times of the imaged tissues. In the next section, we investigate the possibility of finding a rule for ideal imaging parameter identification.

## 4. Optimal Parameter Configuration

After the evaluation of *ρ*, *T*_1_ and *T*_2_ CRLB behaviors, an analysis dedicated to the computation of optimal *T_R_* and *T_E_* combinations is presented. In the following, it will be shown that a proper imaging configuration can greatly improve the performances with respect to such a choice. In particular, the aim of this section is to identify the ideal imaging parameters with respect to imaged tissues.

**Figure 5.**
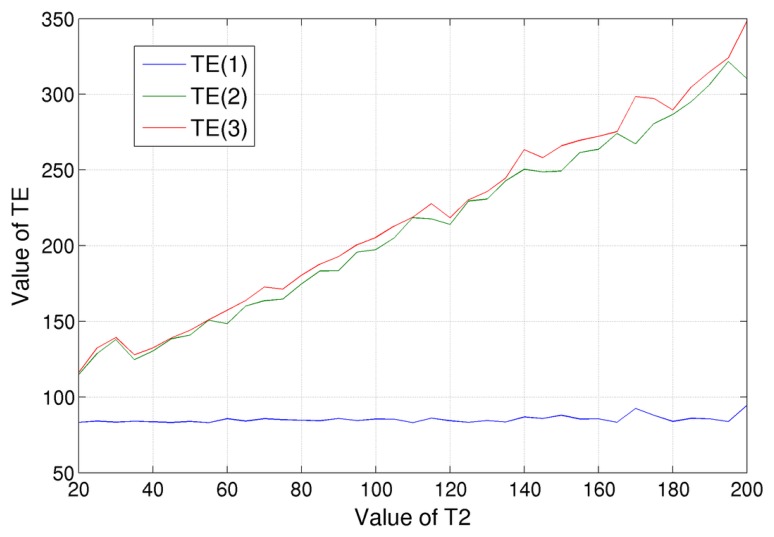
*T_E_* values that minimize the CRLB of *T*_2_ for tissues with different spin-spin relaxation times, *T*_2_. Three values have been considered: the blue line is for the lowest *T_E_* value, the red line for the highest one and green for the intermediate one.

Let us show how the optimal imaging parameters can be determined. Initially, we have focused on the minimization of *T*_2_ CRLB, which consist in finding *T_E_* values that minimize the element (3, 3) of the inverse of the Fisher matrix, **I**(***θ***), of Equation (6) for different spin-spin relaxation times *T*_2_. The optimization has been performed by searching the three optimal *T_E_* values in the [82, 350] ms range for a fixed value of *T_R_*. The evaluation has been done varying the tissue *T*_2_ relaxation times in the [20, 200] ms range, obtaining the results shown in Figure 5. Some considerations can be drawn:

- there is no *T_E_* value combination that is simultaneously ideal for tissues with different spin-spin relaxation times. As a consequence, we can only find the *T_E_* combination that is ideal for a specific tissue;
- by analyzing Figure 5, it can be noticed that the lowest *T_E_* value of the ideal configuration is always equal to the lower bound of the considered variability range, which, in our case, was fixed to 82 ms. As stated before, this value is the minimum echo time for the SE sequence accepted by the Philips Achieva 3.0 T, the MR scanner we worked on;
- the two higher *T_E_* values, which are the red and the green lines of Figure 5, practically coincide. This can be explained considering that we are interested in the estimation of relaxation times, *i.e.*, of decay rates. In order to achieve such a goal, it is crucial that the measurement of the signal decrease, *i.e.*, the ratio of the signal acquired in two different echo times. Therefore, instead of values *T_E_*, it is only important the difference between them. A third echo time, *T_E_*(3), equal to *T_E_*(2), allows us to compute twice the signal decay, which is the quantity in which we are interested;
- the red and the green lines of Figure 5 show a clear trend: their values grow linearly when increasing *T*_2_. In particular, we found that the distance with the blue line (*i.e.*, lowest *T_E_*, 82 ms) is a little bit bigger than the value of the considered spin-spin relaxation time, *T*_2_. For example, in the case of *T*_2_ = 100 ms, the ideal echo times were *T_E_* = [83, 197, 205] ms; the last two values are approximately 110% of (*T_E_*(1) + *T*_2_). By considering the other simulated *T*_2_ values, we found that this coefficient is 110% ± 10%. Within this range, the CRLB of *T*_2_ can be considered constant.

From these simulations, we can derive an empirical rule for the optimal *T_E_* selection: the lower one should be fixed to the minimum value accepted by the MR scanner, while the other values should to be set in the range of 100%–120% of the value of (*T_E_*(1) + *T*_2_), considering the *T*_2_ of the tissue in which we are interested.

**Figure 6.**
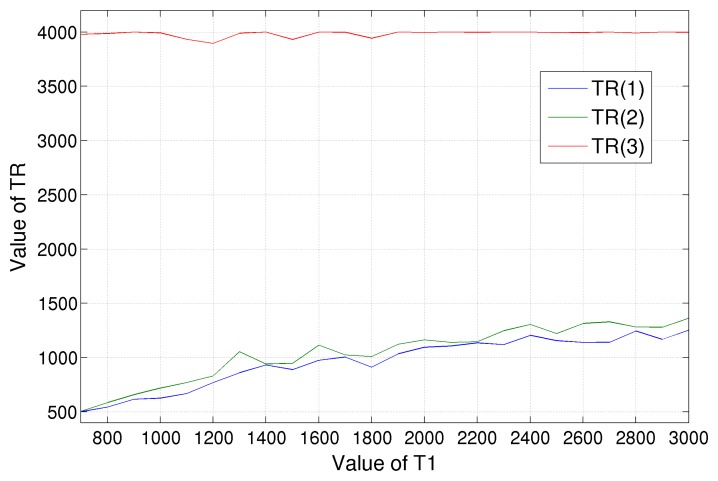
*T_R_* values that minimize the CRLB of *T*_1_ for tissues with different spin-lattice relaxation times, *T*_1_. Three values have been considered: the red line is for the highest *T_R_* value, the blue line for the lowest one and green for the intermediate one.

A similar evaluation has been conducted for the minimization of *T*_1_ CRLB varying MRI scanner repetition times *T_R_*, with a fixed value of *T_E_*; the results are shown in Figure 6. The higher *T_R_* value is fixed to the right edge of the considered variability range, which we set equal to 4, 000 ms. The intermediate and low *T_R_*s have similar values, which, starting from 500 ms in the case of tissue with *T*_1_ = 700 ms, grow almost linearly up to 1, 400 ms for tissues with higher *T*_1_ (about 3, 000 ms). It is hard to determine an empirical rule in this case; anyway, we can say that a choice of around 1, 000 ms for *T_R_*(1) and *T_R_*(2) will fit a wide class of tissues, *i.e.*, those with 1, 200 < *T*_1_ < 2, 000 ms.

**Figure 7.**
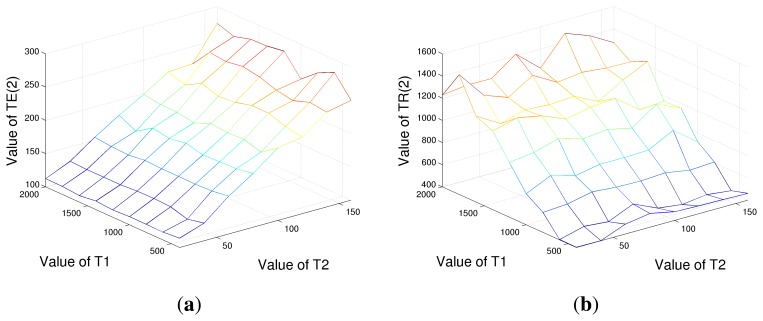
Optimal *T_E_*(2) (**a**) and *T_R_*(2) (**b**) values considering nine acquisitions in the case of tissues with different *T*_1_ and *T*_2_ relaxation times. It can be noticed that the *T_E_*(2) value is substantially independent from tissue spin-lattice relaxation time *T*_1_, as far as *T_R_*(2) from spin-spin relaxation time *T*_2_.

Concluding this section, one more evaluation has been conducted. Instead of optimizing *T_R_* and *T_E_* values separately, a joint minimization has been done. Nine acquisitions have been considered, related to three repetition and three echo times. Among the three values, the lower and the higher have been fixed to the search range bounds, so only the intermediate *T_E_* and *T_R_* values were variable. Results are shown in Figure 7, respectively. It is evident from the figure that *T_E_* values can be considered independent from *T*_1_, as far as *T_R_* from *T*_2_, proving the correctness of the separate optimization of the echo and repetition times. In particular, from Figure 7a, we can state that tissues with equal *T*_2_, but very different *T*_1_ values share the same three optimal echo times for *T*_2_ estimation, and *vice versa*. That said, the behaviors of Figures 5 and 6, *i.e.*, the minimization, one parameter at a time, are confirmed.

In order to easily apply the obtained results, the ideal acquisition parameters for different tissues have been computed exploiting CRLB minimization in the case of a 1.5 T and a 3 T MRI scanner. The results are shown in Tables 1 and 2 for *T*_1_ and *T*_2_, respectively. According to the results reported in Figure 7, the minimizations have been computed independently for spin-lattice and spin-spin relaxation time estimation. Tissue relaxation times have been simulated according to reference values present in the literature [25], which are reported in Table 3.

In Table 4, optimal echo times in the case of gray matter *T*_2_ estimation for different minimum *T_E_* are reported. It can be noticed that the lower optimal echo time is always the minimum and that the empirical rule is confirmed.

Note that the usefulness of a proper *T_R_* and *T_E_* selection, besides the lower estimation variance, consists also in reducing the acquisition time. In order to make such an advantage evident, Table 5 reports the achievable performance in the case of 16 images (4 *T_R_* and 4 *T_E_* values) when moving from equally spaced to optimized acquisition parameters. In particular, the last column of Table 5 shows that 12 acquisitions, with properly chosen parameters, can lead to better results with respect to 16 equally spaced images, while definitely reducing the global acquisition time.

## 5. Numerical Experiments

Within this section, some numerical results are shown in order to validate the advantage of the optimal selection of the imaging parameters according to the previously reported theoretical studies. A tissue with parameters [*ρ T*_1_ *T*_2_] = [5.5 775 44.5] has been considered. Three noisy datasets (SNR = 30 dB) have been simulated, each one composed of four acquisitions. The parameters of Dataset 1 have been chosen according to the results of Figures 5 and 6 in order to optimize the estimation for the considered tissue. Datasets 2 and 3 have been generated with non-ideal parameters. The dataset characteristics are summarized in Table 6.

To asses and validate the CRLB studies, the estimation of the relaxation times has been implemented via Monte Carlo simulation. In particular, a maximum likelihood estimator (MLE) has been set up in the complex domain. Considering that the noise is circularly Gaussian distributed, MLE corresponds to a non-linear least squares (NLLS) estimator [18]. It is important to note that the previously reported theoretical studies about the optimal selection of the imaging parameters are valid for any unbiased estimator, since CRLBs are related only to the acquisition model. Among different estimators, NLLS has been chosen thanks to its low computational times and complexity. We recall that the choice of the optimal estimator is not the aim of this paper.

**Figure 8.**
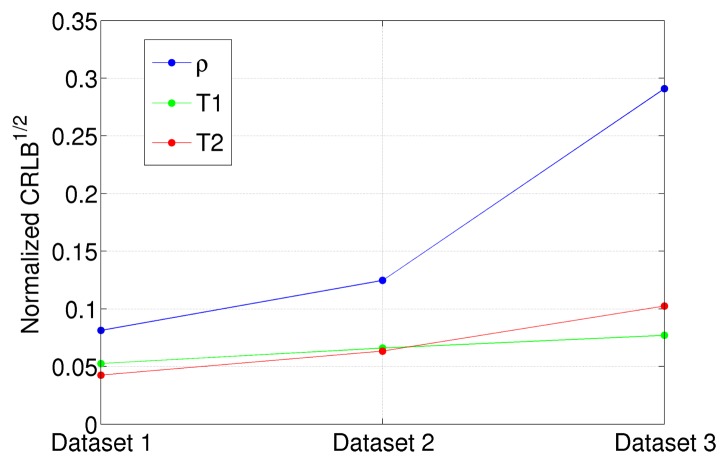
Square root of the CRLB for proton density (blue), spin-lattice (*T*_1_) relaxation time (green) and spin-spin (*T*_2_) relaxation time (red) for the dataset with different acquisition parameters. CRLBs values have been normalized for the parameter values in order to be plotted in the same graph.

The NLLS estimator for the *ρ*, *T*_1_ and *T*_2_ parameters has been implemented on the three datasets of Table 6. A quantitative analysis of the results, in terms of estimation means and variances, has been reported in Table 7. By analyzing it, it is possible to infer that the estimator means are very close, while variances significantly vary from one dataset to the other. In particular, the smallest variances are obtained in the case of Dataset 1. This fully agrees with the theoretical studies reported in Section 4; as a matter of fact, Dataset 1 has been generated by using the previously developed optimal *T_E_* and *T_R_* parameter selection for the considered relaxation times. It is evident that choosing a non-ideal imaging parameters configuration can lead to very inaccurate results. For example, the *T*_2_ estimator variance of Dataset 3 is approximately six times larger than the one of Dataset 1. In order to visualize such results, the normalized standard deviations of *ρ*, *T*_1_ and *T*_2_ in the case of Datasets 1, 2 and 3 are reported in Figure 8.

**Figure 9.**
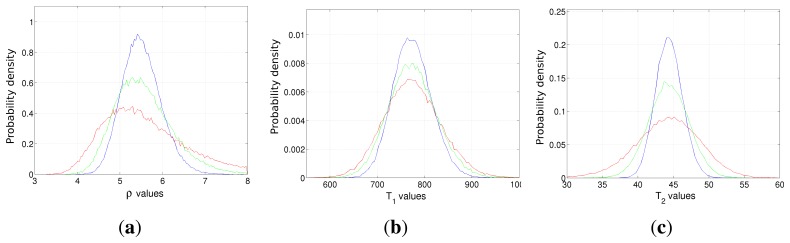
The empirical probability density function of the *ρ* (**a**), *T*_1_ (**b**) and *T*_2_ (**c**) estimators in the case of Dataset 1 (blue line), Dataset 2 (green line) and Dataset 3 (red line). The true values are *ρ* = 5.5, *T*_1_ = 775 and *T*_2_ = 44.5, respectively.

The higher achievable accuracy in the case of optimal imaging parameters selection can also be inferred from the empirical probability density functions of the estimators, reported in Figure 9. In each image, the blue, the green and the red curves refer to Datasets 1, 2 and 3 of Table 6. As expected, most of the presented estimators follow a Gaussian distribution, with a different width. Blue curves obtained using Dataset 1, characterized by the optimal *T_R_*/*T_E_* values for the simulated tissue, are always the narrowest (smallest variances). Moving to curves obtained from Datasets 2 and 3, the estimation error becomes larger. Moreover, in the case of the *ρ* estimator, the results start showing a bias in the case of Dataset 3, *i.e.*, the one with the *worst* acquisition parameters, and the empirical PDF does not look like a Gaussian function any more.

Finally, one further simulation is presented. Signals from two different tissues have been simulated, with parameters [*ρ T*_1_ *T*_2_] = [5 700 68] (spinal cord) and [*ρ T*_1_ *T*_2_] = [5 1190 115] (gray matter). Two datasets composed of four acquisitions have been generated, with parameters reported in Table 8. Taking into account the developed procedure, Dataset 1 parameters represent the ideal configuration for the first tissue, while Dataset 2 is the ideal for the second one.

**Figure 10.**
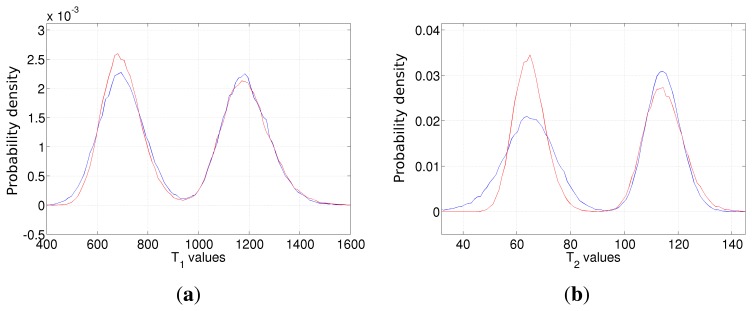
The empirical probability density function of the *T*_1_ (**a**) and *T*_2_ (**b**) estimators in the case of Dataset 1 (blue line) and Dataset 2 (red line). Dataset 1 (blue line) imaging parameters are ideal for tissues with lower *T*_1_ and *T*_2_. On the contrary, Dataset 2 (red line) is ideal for the tissue with higher relaxation times.

The empirical probability density functions for *T*_1_ and *T*_2_ estimators have been computed for both datasets and are shown in Figures 10, respectively. Once again, The results validate the theoretical study of Section 4. Estimation based on Dataset 1 (red line) shows lower variance in the case of spinal cord, *i.e.*, the tissue with the lowest relaxation times (the left peaks of Figures 10). Considering gray matter, Dataset 2 (blue line) -based estimation gives better results, although the improvement of the *T*_1_ estimator is not pronounced. Once again, the result highlights the need of properly tuning the acquisition parameters.

## 6. Conclusions

Within this paper, an analysis on the spin echo signal model in MR imaging has been addressed. In particular, Cramer–Rao lower bounds for relaxation time estimation in the case of a complex Gaussian acquisition model have been evaluated. Several CRLB-based evaluations have been presented in order to investigate the possibility of finding the optimal, in terms of reconstruction accuracy, imaging parameter configuration for the estimation of *T*_1_ and *T*_2_ maps. According to these theoretical studies, an empirical rule together with the identification of the optimal imaging parameter combination (echo and repetition times) in case of different tissues (different *T*_1_ and *T*_2_) has been proposed. Moreover, the optimal acquisition parameters for several tissues have been computed for both 1.5 T and 3 T acquisitions. The theoretical results have been numerically validated on different datasets. It should be underlined that such optimal parameters are independent from the implemented estimators, as CRLBs only depend on the signal model. Once the data have been acquired, different estimators proposed in the literature can be applied. It is important to underline that the theoretical studies reported within the paper can be easily adapted to different imaging sequences.

## Figures and Tables

**Table 1.** Optimal repetition times, *T_R_*, for *T*_1_ estimation in case of different tissues and numbers of acquisitions at 1.5 T and 3 T.

**Tissue**	**1.5T**			**3T**		
	
	**2 images**	**3 images**	**4 images**	**2 images**	**3 images**	**4 images**
liver	490; 4000	490; 510; 4,000	380; 490; 510; 4,000	650; 4,000	570; 650; 4,000	570; 570; 650; 4,000
skeletal muscle	720; 4,000	720; 720; 4,000	680; 720; 720; 4,000	1,090; 4,000	990; 1,090; 4,000	870; 990; 1,090; 4,000
heart	840; 4,000	770; 840; 4,000	770; 790; 840; 4,000	1,060; 4,000	910; 1,060; 4,000	770; 910; 1,060; 4,000
kidney	570; 4,000	430; 570; 4,000	430; 460; 570; 4,000	910; 4,000	790; 910; 4,000	750; 790; 910; 4,000
cartilage	760; 4,000	690; 760; 4,000	630; 690; 760; 4,000	880; 4,000	770; 880; 4,000	770; 780; 880; 4,000
white matter	690; 4,000	690; 710; 4,000	640; 690; 710; 4,000	850; 4,000	780; 850; 4,000	730; 780; 850; 4,000
gray matter	920; 4000	840; 920; 4000	800; 840; 920; 4,000	1,150; 4,000	980; 1,150; 4,000	910; 980; 1,150; 4,000
optic nerve	960; 4,000	960; 1,060; 4,000	960; 1,060; 1,100; 4,000	970; 4,000	910; 970; 4,000	910; 970; 1,030; 4,000
spinal cord	600; 4,000	550; 600; 4,000	450; 550; 600; 4,000	760; 4,000	660; 760; 4,000	660; 700; 760; 4,000
blood	1,120; 4,000	840; 1,120; 4,000	830; 840; 1,120; 4,000	1,120; 4,000	1,040; 1,120; 4,000	1,030; 1,040; 1,120; 4,000

**Table 2.** Optimal echo times *T_E_* for *T*_2_ estimation in case of different tissues and numbers of acquisitions at 1.5 T and 3 T.

**Tissue**	**1.5T**			**3T**		
	
	**2 images**	**3 images**	**4 images**	**2 images**	**3 images**	**4 images**
liver	82; 134	82; 134; 138	82; 134; 138; 146	82; 134	82; 134; 134	82; 134; 134; 142
skeletal muscle	82; 130	82; 130; 138	82; 130; 138; 1;400	82; 132	82; 132; 144	82; 132; 144; 146
heart	82; 124	82; 124; 134	82; 124; 134; 136	82; 132	82; 132; 140	82; 132; 140; 148
kidney	82; 158	82; 158; 168	82; 158; 168; 188	82; 144	82; 144; 154	82; 144; 154; 164
cartilage	82; 116	82; 116; 116	82; 116; 116; 122	82; 114	82; 112; 114	82; 112; 114; 120
white matter	82; 162	82; 162; 188	82; 162; 188; 208	82; 162	82; 162; 178	82; 162; 178; 214
gray matter	82; 210	82; 210; 244	82; 210; 244; 280	82; 192	82; 192; 218	82; 192; 218; 240
optic nerve	82; 192	82; 192; 222	82; 192; 222; 250	82; 168	82; 168; 196	82; 168; 196; 240
spinal cord	82; 160	82; 160; 192	82; 160; 192; 208	82; 174	82; 174; 190	82; 174; 190; 212
blood	82; 516	82; 516; 558	82; 516; 558; 620	82; 436	82; 436; 562	82; 436; 562; 588

**Table 3.** Mean spin-lattice and spin-spin relaxation times for the considered tissues at 1.5 T and 3 T.

**Tissue**	**1.5T**		**3T**	
	
	***T*_1_**	***T*_2_**	***T*_1_**	***T*_2_**
liver	576	46	818	42
skeletal muscle	1,008	44	1,412	50
heart	1,030	40	1,471	47
kidney	690	55	1,194	56
cartilage	1,038	44	1,156	43
white matter	884	72	1,084	69
gray matter	1,124	95	1,820	99
optic nerve	815	77	1,083	78
spinal cord	745	74	993	78
blood	1,441	290	1,932	275

**Table 4.** Optimal echo times for *T*_2_ estimation of gray matter for acquisitions at 1.5 T in the case of different minimum *T_E_* values.

	**2 images**	**3 images**	**4 images**
minimum *T_E_* = 82 ms	82, 210	82, 210, 244	82, 210, 244, 280
minimum *T_E_* = 50 ms	50, 182	50, 182, 212	50, 182, 212, 234
minimum *T_E_* = 20 ms	20, 158	20, 158, 180	20, 158, 180, 210

**Table 5.** CRLBs for equally and optimally spaced *T_R_* and *T_E_* values.

**Tissue parameter**	**CRLB: 16 images Equispaced**	**CRLB: 16 images Optimized**	**Improvement (**%**)**	**CRLB: 12 images Optimized**	**Improvement (**%**)**
*ρ*	0.1562	0.1291	17.34%	0.1506	3.58%
*T*_1_	3144	1483	52.83%	1.960	37.66%
*T*_2_	2.708	1.808	33.23%	2.144	20.83%

**Table 6.** Acquisition parameters: three datasets composed of four images.

	**Repetition Times (s)**	**Echo Times (ms)**	**SNR (dB)**
Dataset 1	0.55, 4.0	80, 140	30
Dataset 2	0.75, 4.0	80, 170	30
Dataset 3	0.90, 4.0	80, 200	30

**Table 7.** Estimator performances: three datasets composed of four images.

**Parameter**	**True Value**	**Dataset 1**		**Dataset 2**		**Dataset 3**	

		**Mean**	**Variance**	**Mean**	**Variance**	**Mean**	**Variance**
*ρ̂*	5.5	5.52	0.20	5.58	0.47	5.79	2.56
*T̂*_1_	775	776.1	1662	776.5	2615	776.5	3, 566
*T̂***_2_**	44.5	44.54	3.58	44.49	7.95	44.32	20.82

**Table 8.** Acquisition parameters in the case of two tissues; the datasets are composed of four images.

	**Repetition Times (s)**	**Echo Times (ms)**	**SNR (dB)**
Dataset 1	0.55 4.0	82, 160	26
Dataset 2	0.80, 4.0	82, 250	26

## Appendix

From [16], CRLBs may also be expressed in a slightly different form with respect to Equation (6). In particular, it yields:

(7) $$-E\;\left[\frac{\partial^{2}\ln\;p(\text{y};\theta )}{\partial \theta^{2}}\right]=E\;\left[{\left(\frac{\partial \ln\;p(y;\theta )}{\partial \theta }\right)}^{2}\right]$$

From Equation (4), the log-likelihood function related to *N* complex acquisitions is:

$$\log[p(y;\theta )]=-N\log(2\pi \sigma^{2})-\frac{1}{2\sigma^{2}}\sum_{k=1}^{N}\left[f^{2}(\theta )+y_{R}^{2}(k)+y_{I}^{2}(k)-2f(\theta )y_{R}(k)\cos(\phi )-2f(\theta )y_{I}(k)\sin(\phi )\right]$$

where subscript *k* refers to *k*-th acquisition, *i.e.*, the MRI scan with parameters *T_R_*(*k*), *T_E_*(*k*). The partial derivatives can be computed as:

$$\begin{array}{ll} \frac{\partial \ln\;p(\text{y};\theta )}{\partial \rho } & =-\frac{1}{2\sigma^{2}}\overset{N}{\underset{k=1}{\text{∑}}}\left[\frac{\partial f^{2}(\theta )}{\partial \rho }-2\frac{\partial f(\theta )}{\partial \rho }(y_{R}(k)\cos(\phi )+y_{I}(k)\sin(\phi ))\right]\\ \frac{\partial \ln\;p(y;\theta )}{\partial T_{1}} & =-\frac{1}{2\sigma^{2}}\overset{N}{\underset{k=1}{\text{∑}}}\left[\frac{\partial f^{2}(\theta )}{\partial T_{1}}-2\frac{\partial f(\theta )}{\partial T_{1}}(y_{R}(k)\cos(\phi )+y_{I}(k)\sin(\phi ))\right]\\ \frac{\partial \ln\;p(y;\theta )}{\partial T_{2}} & =-\frac{1}{2\sigma^{2}}\overset{N}{\underset{k=1}{\text{∑}}}\left[\frac{\partial f^{2}(\theta )}{\partial T_{2}}-2\frac{\partial f(\theta )}{\partial T_{2}}(y_{R}(k)\cos(\phi )+y_{I}(k)\sin(\phi ))\right] \end{array}$$

where the first order derivatives are:

$$\begin{array}{ll} \frac{\partial f(\theta )}{\partial \rho } & =\exp\;\left(-\frac{T_{E}}{T_{2}}\right)\;\left[1-\exp\;\left(\frac{T_{R}}{T_{1}}\right)\right]\\ \frac{\partial f^{2}(\theta )}{\partial \rho } & =2\rho \exp\;\left(-\frac{2T_{E}}{T_{2}}\right)\;{\left[1-\exp\;\left(\frac{T_{R}}{T_{1}}\right)\right]}^{2}\\ \frac{\partial f(\theta )}{\partial T_{1}} & =-\rho \frac{T_{R}}{T_{1}^{2}}\exp\;\left(-\frac{T_{E}}{T_{2}}\right)\exp\;\left(-\frac{T_{R}}{T_{1}}\right)\\ \frac{\partial f^{2}(\theta )}{\partial T_{2}} & =-2\rho^{2}\frac{T_{R}}{T_{1}^{2}}\exp\;\left(-\frac{2T_{E}}{T_{2}}\right)\exp\left(-\frac{T_{R}}{T_{1}}\right)\;\left[1-\exp\left(-\frac{T_{R}}{T_{1}}\right)\right]\\ \frac{\partial f(\theta )}{\partial T_{2}} & =\rho \frac{T_{E}}{T_{2}^{2}}\exp\;\left(-\frac{T_{E}}{T_{2}}\right)\;\left[1-\exp\left(\frac{T_{R}}{T_{1}}\right)\right]\\ \frac{\partial f^{2}(\theta )}{\partial T_{2}} & =2\rho^{2}\frac{T_{E}}{T_{2}^{2}}\exp\;\left(-\frac{2T_{E}}{T_{2}}\right)\;{\left[1-\exp\;\left(\frac{T_{R}}{T_{1}}\right)\right]}^{2} \end{array}$$

In order to compute the expected value of Equation (7), Monte Carlo simulations have to be implemented.
